# Accuracy of Fitbit Charge 4, Garmin Vivosmart 4, and WHOOP Versus Polysomnography: Systematic Review

**DOI:** 10.2196/52192

**Published:** 2024-03-27

**Authors:** An-Marie Schyvens, Nina Catharina Van Oost, Jean-Marie Aerts, Federica Masci, Brent Peters, An Neven, Hélène Dirix, Geert Wets, Veerle Ross, Johan Verbraecken

**Affiliations:** 1 Multidisciplinary Sleep Disorders Centre, Antwerp University Hospital, Edegem, Belgium; 2 Laboratory of Experimental Medicine and Pediatrics, University of Antwerp, Wilrijk, Belgium; ^3^Department of Biosystems, KU Leuven, Leuven, Belgium; 4 Transportation Research Institute (IMOB), School of Transportation Sciences, UHasselt, Hasselt, Belgium; 5 Faresa, Evidence-Based Psychological Centre, Hasselt, Belgium

**Keywords:** sleep, wearable device, validation, polysomnography, assessing sleep, PRISMA, Preferred Reporting Items for Systematic Reviews and Meta-Analyses

## Abstract

**Background:**

Despite being the gold-standard method for objectively assessing sleep, polysomnography (PSG) faces several limitations as it is expensive, time-consuming, and labor-intensive; requires various equipment and technical expertise; and is impractical for long-term or in-home use. Consumer wrist-worn wearables are able to monitor sleep parameters and thus could be used as an alternative for PSG. Consequently, wearables gained immense popularity over the past few years, but their accuracy has been a major concern.

**Objective:**

A systematic review of the literature was conducted to appraise the performance of 3 recent-generation wearable devices (Fitbit Charge 4, Garmin Vivosmart 4, and WHOOP) in determining sleep parameters and sleep stages.

**Methods:**

Per the PRISMA (Preferred Reporting Items for Systematic Reviews and Meta-Analyses) statement, a comprehensive search was conducted using the PubMed, Web of Science, Google Scholar, Scopus, and Embase databases. Eligible publications were those that (1) involved the validity of sleep data of any marketed model of the candidate wearables and (2) used PSG or an ambulatory electroencephalogram monitor as a reference sleep monitoring device. Exclusion criteria were as follows: (1) incorporated a sleep diary or survey method as a reference, (2) review paper, (3) children as participants, and (4) duplicate publication of the same data and findings.

**Results:**

The search yielded 504 candidate articles. After eliminating duplicates and applying the eligibility criteria, 8 articles were included. WHOOP showed the least disagreement relative to PSG and Sleep Profiler for total sleep time (−1.4 min), light sleep (−9.6 min), and deep sleep (−9.3 min) but showed the largest disagreement for rapid eye movement (REM) sleep (21.0 min). Fitbit Charge 4 and Garmin Vivosmart 4 both showed moderate accuracy in assessing sleep stages and total sleep time compared to PSG. Fitbit Charge 4 showed the least disagreement for REM sleep (4.0 min) relative to PSG. Additionally, Fitbit Charge 4 showed higher sensitivities to deep sleep (75%) and REM sleep (86.5%) compared to Garmin Vivosmart 4 and WHOOP.

**Conclusions:**

The findings of this systematic literature review indicate that the devices with higher relative agreement and sensitivities to multistate sleep (ie, Fitbit Charge 4 and WHOOP) seem appropriate for deriving suitable estimates of sleep parameters. However, analyses regarding the multistate categorization of sleep indicate that all devices can benefit from further improvement in the assessment of specific sleep stages. Although providers are continuously developing new versions and variants of wearables, the scientific research on these wearables remains considerably limited. This scarcity in literature not only reduces our ability to draw definitive conclusions but also highlights the need for more targeted research in this domain. Additionally, future research endeavors should strive for standardized protocols including larger sample sizes to enhance the comparability and power of the results across studies.

## Introduction

Sleep problems have emerged as a widespread concern with implications on health and quality of life for many people worldwide [1]. It has been suggested that 67% of adults worldwide have sleep problems [2,3]. The amount and quality of sleep that someone enjoys have a lasting impact during wakefulness. It affects mental health; physical well-being; and even the risk of developing lifestyle diseases such as cardiovascular diseases, obesity, depression, and type 2 diabetes [4-7]. Considering that sleep is vital to our health and quality of life, it is reasonable to wonder how long someone actually sleeps each night and if someone is getting enough restful and restorative sleep to keep the body and mind in optimal condition. Hence, this is why sleep tracking has gained immense popularity over the past few years. The majority of sleep trackers provide data on sleep architecture and hypnograms through their associated apps, offering insights into sleep stages and patterns [8]. In addition, these wearables can notify you about specific factors that might be affecting your sleep patterns such as drinking water, exercise, meditation, and regular bedtimes. As such, they can be a useful tool to obtain more insights into sleeping habits and patterns and to help optimize your sleep hygiene and quality. However, to improve sleep, an accurate, objective measurement is mandatory.

Polysomnography (PSG) is the gold-standard method for objectively assessing sleep. PSG records signals of brain activity, eye movements, and muscle tone, as well as audio and video, enabling it to classify sleep stages [9]. However, PSG may not be ideal for monitoring sleep in particular settings, as it is expensive, labor-intensive, and time-consuming; requires various equipment and technical expertise; and is impractical for long-term use or in-home environment settings [10,11]. PSG involves real-time monitoring of various parameters. In addition, applying and removing the sensors, organizing the patient administration, and thoroughly analyzing the data that PSG add is quite labor-intensive for sleep technicians. In addition to applying and removing the sensors and possibly completing questionnaires and other administrative tasks, PSG requires an overnight stay at a sleep clinic or laboratory, which makes PSG time-consuming for both the sleep technicians and patients. Due to the inherent limitations of PSG, several alternatives have been proposed. First, the use of sleep diaries is inexpensive and straightforward for consumers, but the subjective self-ratings they require result in frequent inaccuracies and incompleteness [12]. Additionally, they fail to measure sleep architecture and stages. Second, electroencephalogram (EEG) wearables can provide a home evaluation of sleep architecture and stages; however, they come with a high cost and can be technologically complex [12]. Therefore, wearables using an accelerometer and photoplethysmography (PPG) are being explored as a feasible alternative, largely due to their lower cost, convenience, and ability to measure sleep in clinical and personal settings [13].

Accelerometers and PPG sensors monitor different physiological and movement patterns throughout the night. Accelerometers are small, electromechanical devices that measure acceleration along multiple axes (usually 3: x, y, and z) to detect position changes, turning over, or significant body movements during the night [14]. Due to the body movement variations specific to each sleep stage, accelerometers can provide information about wakefulness and general sleep stages. However, they may tend to overestimate sleep due to poorly distinguishing between sleep and sedentary supine wake periods (eg, lying down while reading or watching television), or they could underestimate sleep due to potential body movements during sleep being categorized as awakenings [12,15-18]. In addition, they may not be as accurate in distinguishing between different non–rapid eye movement (REM) stages and detecting subtle changes in sleep architecture [14]. By combining an accelerometer with a PPG sensor in wearables, a more comprehensive and accurate assessment of sleep could be provided. PPG sensors are a noninvasive technology that uses a light source and a photodetector at the surface of the skin to measure the volumetric variations of blood circulation and thus can be used to monitor heart rate, heart rate variability, blood flow, and blood oxygen levels [19-21]. Due to the specific cardiovascular features of each sleep stage, PPG can provide more information about the sleep stages in addition to the accelerometer [21,22]. The benefits of these sensors used in wearables are their low-cost, noninvasive nature and their ability to provide continuous monitoring and real-time data. However, the readings of PPG can be affected by motion artifacts, skin pigmentation, or tissue thickness. In addition, they could be susceptible to environmental factors such as ambient light and temperature [23-25].

Although many have doubts about their accuracy in monitoring sleep, wearable sleep-tracking devices are widely used and becoming more technologically advanced, creating strong interest from researchers and clinicians for their possible use as alternatives to PSG. This was demonstrated by Nguyen et al [26], who used wearables that provide inactivity alerts and personal feedback to increase physical activity and improve sleep for survivors of breast cancer [26].

Given the disadvantages of PSG and the corresponding growing popularity of wearable devices for sleep tracking among consumers and medical organizations, the objective of this paper was to appraise the performance of recent-generation wearable devices in determining sleep parameters and sleep stages through a review of relevant publications. To limit the overwhelming amount of wearables and their corresponding research papers, we performed a search to select a limited number of recent, frequently used wearables, using the following criteria: recent generation; good ease of use (affordable, unobtrusive, and sufficient battery life); and assessment of variables that could also be used for monitoring sleep, stress, fatigue, and sleepiness—namely, heart rate, heart rate variability, stress indicator, and activity. The candidate wearables selected up-front were Fitbit Charge 4, Garmin Vivosmart 4, and WHOOP.

## Methods

### Search Strategies

**Figure 1.**
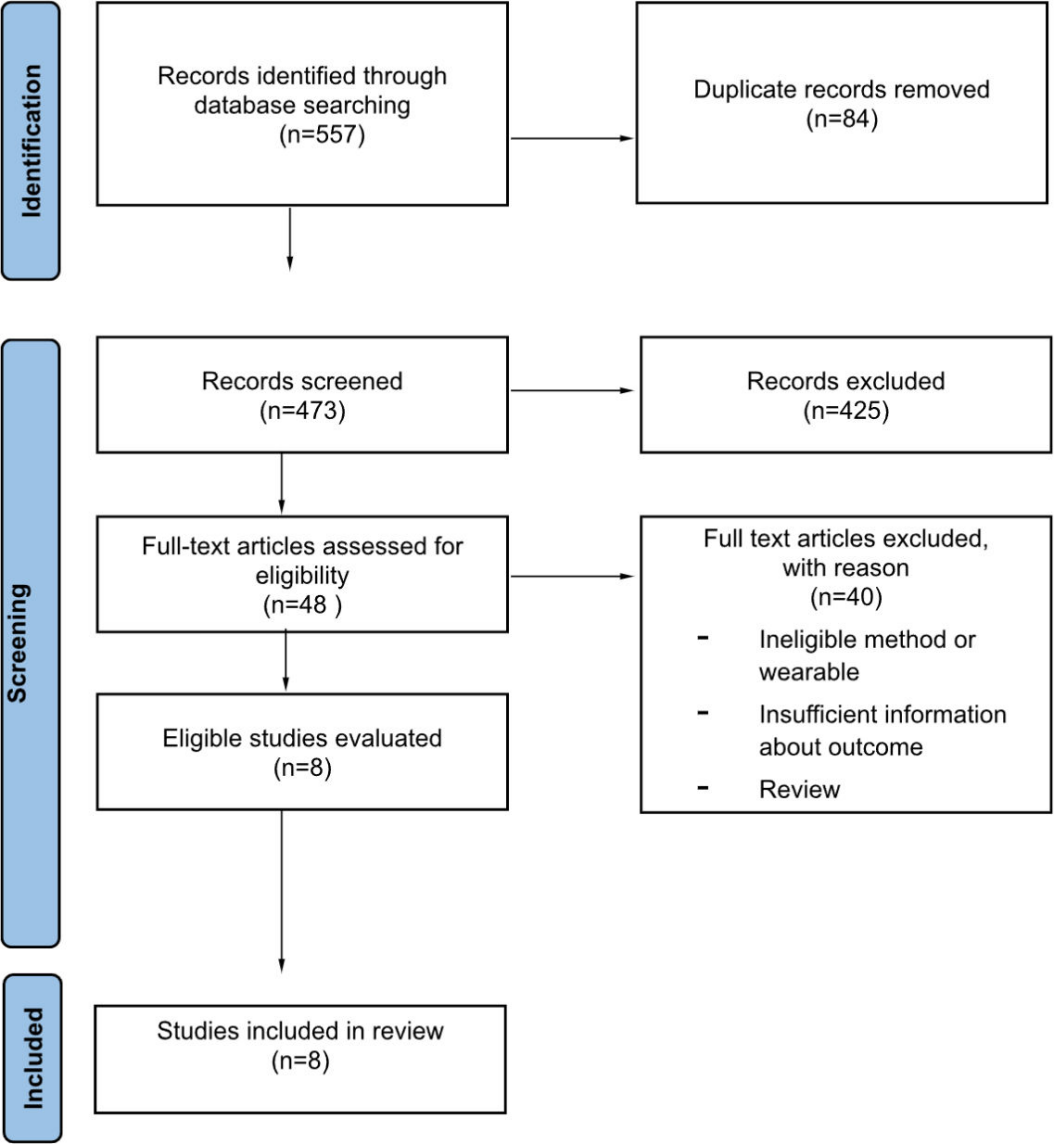
PRISMA (Preferred Reporting Items for Systematic Reviews and Meta-Analyses) flow diagram describing the search strategy of databases to retrieve and qualify publications of relevance for this systematic literature review.

In adherence with the PRISMA (Preferred Reporting Items for Systematic Reviews and Meta-Analyses) statement (Checklist 1), a comprehensive search using the PubMed, Web of Science, Google Scholar, Scopus, and Embase databases was conducted [27]. Relevant keywords such as “validity,” “accuracy,” “assessment,” “performance,” “wearable,” “sleep tracker,” “sleep-tracking,” “polysomnography,” “wristband,” “Whoop,” “Fitbit Charge 4,” and “Garmin Vivosmart 4” were used (see Multimedia Appendix 1). This search was initially completed by May 16, 2023, and was repeated by November 23, 2023 (see Figure 1). This systematic literature review was not registered and a protocol was not predefined.

### Eligibility Criteria

Retrieved publications qualified for the review if they (1) involved the validity of sleep data of any marketed model of the candidate wearables and (2) incorporated PSG or an ambulatory EEG monitor as a reference sleep monitoring device. Exclusion criteria were as follows: (1) incorporated a sleep diary or survey method as a reference, (2) review paper, (3) children as participants, and (4) duplicate publication of the same data and findings.

### Data Extraction

The following items were extracted: type of sleep tracker; number, gender, and age of participants; number of nights of sleep assessment; reference sleep monitoring device; and study outcomes relative to the denoted reference standard—the precision of measuring the parameters of total sleep time (TST), light sleep (LS), deep sleep (DS), and REM sleep, as well as the sensitivity to sleep (the proportion of correctly classified sleep epochs by the wearable); specificity for sleep (the proportion of correctly classified wake epochs by the wearable); agreement; and if applicable, Cohen κ for multistate categorization of sleep periods. These Cohen κ values are a measure of interdevice reliability, often used in the context of validation studies where 2 or more methods of devices are used to assess a particular characteristic or condition [28]. In the case of wearable validation studies for sleep, κ values are often used to assess the agreement between the wearable device’s sleep detection algorithm and a reference standard, such as PSG. In addition to the observed agreement between the devices, κ values also take into account the possibility that the agreement comes by chance. The interpretation of κ values is often categorized as follows: values ≤0 indicate no agreement, 0.01-0.20 indicate none to slight agreement, 0.21-0.40 indicate fair agreement, 0.41-0.60 indicate moderate agreement, 0.61-0.80 indicate substantial agreement, and 0.81-1.00 indicate almost perfect agreement [28].

## Results

### Overview of Included Studies

Figure 1 presents a visual summary of the selection and qualification of articles for this review. A total of 8 publications were retrieved through a search of databases performed by May 2023 and again by November 2023. Table 1 presents an overview of all included studies and the extracted details of each qualifying study involving the different wearable models. Few papers that met the eligibility criteria for the candidate wearables were found; we found 3 papers that satisfy the criteria for Fitbit Charge 4 [28-30], 2 for Garmin Vivosmart 4 [27,28], and 4 for WHOOP [22,30-32].

**Table 1.** Summary table of included papers in this systematic literature review.

Author (year)	Wearable	Reference	Participants, n	Sex, n	Age (y), mean (SD)	Duration
Doheny et al [33] (2021)[Bibr R32][Bibr R32]	Fitbit Charge 4	PSG^a^	2	N/A^b^	N/A	1 night, laboratory based; followed by 7 nights at home
Renerts et al [34] (2022)	Fitbit Charge 4	PSG	8	N/A	N/A	1 night, laboratory based
Dong et al [18] (2022)	Fitbit Charge 4	PSG	37	Female: 20 Male: 17	48.8 (2.1)	1 night, laboratory based
Mouritzen et al [29] (2020)[Bibr R27][Bibr R27]	Garmin Vivosmart 4	PSG	18	Female: 13 Male: 5	56.1 (12.0)	1 night, laboratory based
Stone et al [30] (2020)[Bibr R28][Bibr R28]	Garmin Vivosmart 4 and WHOOP	Sleep Profiler(ambulatory EEG^c^ monitor)	5	Female: 3 Male: 2	27.8 (7.6)	Home environment,98 nights for all study devices
Miller et al [31] (2020)[Bibr R29][Bibr R29]	WHOOP	PSG	12	Female: 6 Male: 6	22.9 (3.4)	10 days, laboratory based
Miller et al [32] (2021)[Bibr R30][Bibr R30]	WHOOP	PSG	6	Female: 3 Male: 3	23.0 (2.2)	9 nights, laboratory based
Miller et al [22] (2022)	WHOOP	PSG	53	Male: 27 Female: 26	25.4 (5.9)	1 night, laboratory based

a PSG: polysomnography.

b N/A: not applicable.

c EEG: electroencephalogram.

Participants were diverse: healthy adults as well as participants diagnosed with Huntington disease [33,34] and chronic insomnia [18]. Sample size varied substantially between investigations, from 2 to 53 participants. The average age of the participants was 34 years old. Out of the 8 studies, 6 (75%) were fully conducted in a sleep laboratory [18,22,29,31,32,34], 1 (12%) was conducted in the home environment [30], and 1 (12%) conducted an overnight laboratory-based PSG followed by 7 nights in the home environment [33]. In all, 3 (38%) laboratory-based studies had a duration of more than 1 night [30-32].

### Comparison of Sleep Parameters Assessed by Wearables Versus PSG

#### Fitbit Charge 4

As shown in Table 2, of the 3 Fitbit Charge 4 versus PSG comparisons, 2 (67%) reported an overestimation of TST (5 and 23 minutes), whereas 1 (33%) reported a nonsignificant underestimation of 11 minutes. For LS, 2 (67%) of the 3 comparisons reported a similar significant overestimation (37.5 and 37.7 minutes). For DS, all 3 papers reported an underestimation (4.1, 12.5, and 41.4 minutes). REM sleep was overestimated in 2 (67%) out of 3 papers by 5.2 and 11.5 minutes and underestimated in 1 (33%) paper by 4.7 minutes. Table 3 shows the results for the sensitivity to and specificity for sleep. The sensitivity to sleep was quite high for all 3 papers (89.9%-93.6%). In contrast, the results for specificity for sleep were quite divergent (48.8%, 62.2%, and 73%). The sensitivities to DS and REM sleep could only be extracted from 2 papers: 54% and 96% for DS and 76% and 97% for REM sleep.

**Table 2.** The total sleep time (TST), light sleep (LS), deep sleep (DS), and rapid eye movement (REM) sleep of Fitbit Charge 4, Garmin Vivosmart 4, and WHOOP versus polysomnography.

Wearable and paper		TST (min)		LS (min)		DS (min)		REM sleep (min)	
		Mean (SD)	*P* value	Mean (SD)	*P* value	Mean (SD)	*P* value	Mean (SD)	*P* value
**Fitbit Charge 4**									
	Doheny et al [33]	23 (N/A^a^)	N/A	37.5 (N/A)	N/A	−12.5 (N/A)	N/A	11.5 (N/A)	N/A
	Renerts et al [34]	5 (26.8)	N/A	N/A	N/A	−4.1 (21.8)	N/A	5.21 (22.35)	N/A
	Dong et al [18]	−11.0 (N/A)	.16	37.69 (N/A)	.001	−41.38 (N/A)	<.0001	−4.7 (N/A)	.44
	Mean	5.67 (N/A)	N/A	37.6 (N/A)	N/A	−19.33 (N/A)	N/A	4 (N/A)	N/A
**Garmin Vivosmart 4**									
	Mouritzen et al [29]	27.8 (29.5)	.001	36.5 (71.7)	.045	13.4 (98.1)	.57	−22.1 (54.7)	.11
	Stone et al [30]	66 (N/A)	.06	19.8 (N/A)	1	33.6 (N/A)	1	−3 (N/A)	1
	Mean	46.9 (N/A)	N/A	28 (N/A)	N/A	23.5 (N/A)	N/A	−12.55 (N/A)	N/A
**WHOOP**									
	Stone et al [30]	16.2	.61	−10.2	1	1.8	1	6	1
	Miller et al [31]	8.2 (32.9)	.54	−3.7 (44.4)	.62	−3.7 (26.4)	.33	15.6 (39.7)	.01
	Miller et al [32]	−17.8 (61.1)	N/A	−8.9 (55.9)	<.05	−15.5 (30.1)	<.001	6.5 (39.5)	N/A
	Miller et al [22]	−12.2 (36.3)	N/A	−15.6 (50.7)	N/A	−19.6 (34.3)	N/A	22.9 (45.4)	N/A
	Mean	−1.4 (N/A)	N/A	−9.6 (N/A)	N/A	−9.25 (N/A)	N/A	12.75 (N/A)	N/A

a N/A: not applicable.

**Table 3.** The sensitivity to sleep, specificity for sleep, agreement, and Cohen κ coefficient for multistate categorization of Fitbit Charge 4, Garmin Vivosmart 4, and WHOOP versus polysomnography.

Wearable and paper		Sensitivity to sleep (%), mean (SD)	Specificity for sleep (%), mean (SD)	Sensitivity to light sleep (%), mean (SD)	Sensitivity to deep sleep (%), mean (SD)	Sensitivity to REM^a^ sleep (%), mean (SD)	Agreement (%), mean (SD)	Cohen κ for multistate categorization, mean (SD)
**Fitbit Charge 4**								
	Doheny et al [33]	93.6 (2.6)	48.8 (17.7)	N/A^b^	96 (N/A)	97 (N/A)	N/A	N/A
	Renerts et al [34]	90 (N/A)	73 (N/A)	N/A	54 (N/A)	76 (N/A)	N/A	N/A
	Dong et al [18]	89.9 (4.0)	62.2 (26.2)	N/A	N/A	N/A	N/A	N/A
	Mean	91.2 (N/A)	61.3 (N/A)	N/A	75 (N/A)	86.5 (N/A)	NA	N/A
**Garmin Vivosmart 4**								
	Mouritzen et al [29]	98 (3)	30 (17)	60 (17)	45 (26)	34 (26)	48 (10)	0.20 (0.11)
	Stone et al [30]	N/A	N/A	N/A	N/A	N/A	N/A	N/A
	Mean	98 (3)	30 (17)	60 (17)	45 (26)	34 (26)	48 (10)	N/A
**WHOOP**								
	Stone et al [30]	N/A	N/A	N/A	N/A	N/A	N/A	N/A
	Miller et al [31]	95 (N/A)	51 (N/A)	62 (N/A)	68 (N/A)	70 (N/A)	64 (N/A)	0.47 (N/A)
	Miller et al [32]	90 (N/A)	60 (N/A)	61 (N/A)	64 (N/A)	66 (N/A)	63 (N/A)	0.47 (N/A)
	Miller et al [22]	90 (N/A)	56 (N/A)	58 (N/A)	62 (N/A)	66 (N/A)	60 (N/A)	0.44 (N/A)
	Mean	91.7 (N/A)	56 (N/A)	60 (N/A)	65 (N/A)	67 (N/A)	62 (N/A)	0.46 (N/A)

a REM: rapid eye movement.

b N/A: not applicable.

#### Garmin Vivosmart 4

Both papers comparing Garmin Vivosmart 4 to PSG or Sleep Profiler reported an overestimation of TST (27.8 and 66 minutes), as shown in Table 2. For LS, both papers again reported an overestimation (19.8 and 36.5 minutes). For DS, both papers reported an overestimation (13.4 and 33.6 minutes). On the contrary, REM sleep was underestimated by both papers by 3 and 22.1 minutes. The study from Mouritzen et al [29] reported sensitivities to LS, DS, and REM sleep of 60%, 45%, and 34%, respectively; a sensitivity to sleep of 98%; a specificity for sleep of 30%; an agreement of 48%; and a Cohen κ for multistate categorization of sleep periods of 0.20 (see Table 3).

#### WHOOP

As shown in Table 2, of the 4 WHOOP versus PSG comparisons, 2 (50%) reported an overestimation of TST (8.2 and 16.2 minutes), whereas the other 2 (50%) reported an underestimation (12.2 and 17.8 minutes). All 4 comparisons reported an underestimation of LS, from 3.7 to 15.6 minutes. For DS, 3 (75%) papers reported an underestimation (3.7, 15.5, and 19.6 minutes). Stone et al [30] reported a small overestimation of DS of 1.8 minutes. REM sleep was overestimated in all 4 studies, from 6 to 22.9 minutes. Table 3 shows that the studies from Miller et al [22,31,32] reported sensitivities to LS, DS, and REM sleep ranging from 58% to 62%, from 62% to 68%, and from 66% to 70%, respectively. The studies also reported a sensitivity to sleep ranging from 90% to 95%, a specificity for sleep ranging from 51% to 60%, an agreement ranging from 60% to 64%, and a Cohen κ for multistate categorization of sleep periods ranging from 0.44 to 0.47.

### Comparison of the Mean Values of the Different Wearables

From Tables 2 and 3, the means of the different values extracted from the papers (ie, TST, LS, DS, REM sleep, sensitivity to sleep, specificity for sleep, sensitivity to LS, sensitivity to DS, sensitivity to REM sleep, agreement, and Cohen κ coefficient) were calculated, which are summarized in Table 4. Altogether, WHOOP deviated the least compared to the gold-standard PSG for TST, LS, and DP but showed the highest difference from PSG for REM sleep (ie, a mean overestimation of 21 min).

**Table 4.** Mean differences of the sleep parameters in minutes and means of sensitivities to sleep, specificity for sleep, and agreement assessed by the wearables compared to polysomnography.

Variable	FitbitCharge 4	GarminVivosmart 4	WHOOP
TST^a^ (min)	5.7	46.9	−1.4
LS^b^ (min)	37.6	27.9	−9.6
DS^c^ (min)	−19.2	23.5	−9.3
REM^d^ sleep (min)	4.0	−12.5	21.0
Sensitivity to sleep (%)	91.2	98.0	91.7
Sensitivity to LS (%)	N/A^e^	60.0	60.0
Sensitivity to DS (%)	75.0	45.0	65.0
Sensitivity to REM sleep (%)	86.5	34.0	67.0
Specificity for sleep (%)	61.3	30.0	55.7
Agreement (%)	N/A	48	62
Cohen κ	N/A	0.20	0.46
Papers, n	3	2	4

a TST: total sleep time.

b LS: light sleep.

c DS: deep sleep.

d REM: rapid eye movement.

e N/A: not applicable.

Garmin Vivosmart 4 showed the largest deviations out of the 3 different wearables compared to PSG (ie, a mean overestimation of 46.9 minutes for TST, a mean overestimation of 27.9 minutes for LS, a mean overestimation of 23.5 minutes for DS, and a mean underestimation of 12.5 minutes for REM sleep). Additionally, the sensitivities to LS, DS, and REM sleep were lower compared to those of Fitbit Charge 4 and WHOOP.

The mean values of Fitbit Charge 4 deviated the least from PSG for REM sleep, with a mean overestimation of only 4 minutes. On the contrary, it showed the largest deviation to PSG for LS, with a mean overestimation of 37.6 minutes. For TST and DS, Fitbit Charge 4 showed on average better results than Garmin Vivosmart 4 but worse results than WHOOP, namely, a mean overestimation of 5.7 minutes and a mean underestimation of 19.2 minutes, respectively. The sensitivities to LS, DS, and REM sleep were higher compared to those of Garmin Vivosmart 4 and WHOOP.

## Discussion

PSG is still the gold-standard method to objectively assess sleep. However, PSG is not ideal for monitoring sleep in particular settings and for long-term follow-up. To overcome these limitations, consumer sleep-tracking devices are becoming more widely used and technologically advanced, creating strong interest from researchers and clinicians for their possible application as alternatives to PSG. Since limited research has been performed to validate the different consumer sleep-tracking wearables, we aimed to review the available literature on the selected wearables to determine the most accurate, commercially available wrist-worn device that can be used in a clinical setting for long-term sleep monitoring.

Thus far, at most, only 7 relevant studies investigated the performance of Fitbit Charge 4, Garmin Vivosmart 4, and WHOOP against PSG. The study of Stone et al [30] investigated the performance of both Garmin Vivosmart 4 and WHOOP relative to Sleep Profiler. After reviewing these studies, the results suggest that WHOOP presented the least amount of disagreement relative to PSG and Sleep Profiler for TST, LS, and DS but showed the largest amount of disagreement for REM sleep. Fitbit Charge 4 and Garmin Vivosmart 4 both showed moderate accuracy in assessing sleep stages and TST compared to PSG. Fitbit Charge 4 showed the least amount of disagreement for REM sleep relative to PSG. In addition, Fitbit Charge 4 showed higher sensitivities for LS, DS, and REM sleep compared to Garmin Vivosmart 4 and WHOOP. Garmin Vivosmart 4 showed the lowest sensitivities to LS, DS, and REM sleep compared to Fitbit Charge 4 and WHOOP.

Some of the studies performed evaluations of the accuracy of wearables in detecting sleep by using an epoch-by-epoch analysis. It involves breaking down the continuous stream of sleep data of PSG into discrete time intervals called “epochs” [35]. Afterward, each epoch is compared individually to the corresponding epoch generated by the wearables. The results of the epoch-by-epoch analysis in this review showed high sensitivity to sleep, ranging from 91.2% to 98.0%, but lower specificity for sleep, ranging from 30% to 61%. The low specificity for sleep, or variability in specificity for sleep, is a commonly observed phenomenon in the validation of devices that primarily rely on actigraphy to estimate sleep [15,36,37]. The challenge of accurately separating wake episodes during sleep stems from the similarities in movement between restful wakefulness and sleep. Hence, it can be inferred that devices that have improved their ability to detect wake epochs during sleep have refined their proprietary algorithms to include metrics other than movement, such as heart rate and heart rate variability, in the detection of wakefulness [22].

Furthermore, it is crucial to contextualize the comparison of the wearables’ agreement with that of PSG (see Multimedia Appendix 2), taking into consideration that the scoring of PSG is subject to variability among technicians [38]. As reported by Danker-Hopfe et al [39], the interrater reliability ranges from 86.5% to 97.5% depending on the sleep stage, with an overall accuracy of 81% and a Cohen κ coefficient of 0.7505 (see Table 5). Given this benchmark, Fitbit Charge 4 seems to provide reasonable estimations of multistate sleep. However, it is imperative to acknowledge that achieving the same level of accuracy as PSG may pose a significant challenge for wearables, particularly considering the observed low Cohen κ coefficients of the wearables. The finding of a low κ value indicates poor sleep stage differentiation despite including PPG signals. However, the beneficial role of PPG signals in accelerometer-based sleep tracking remains unilluminated, since we do not know how these signals are processed and applied [29].

**Table 5.** Sleep stage–specific degree of agreement according to American Academy of Sleep Medicine standards [38].

Stage	Agreement (%)	Cohen κ
Overall	81	0.7505
Wake	95.6	0.4608
REM^a^	97.5	0.9054
N3^b^	93.8	0.7285
N2^c^	86.5	0.7188
N1^d^	90.1	0.4608

a REM: rapid eye movement.

b N3: stage 3 non-REM sleep.

c N2: stage 2 non-REM sleep.

d N1: stage 1 non-REM sleep.

On a similar note, companies usually do not share the methodology they use to score the sleep data from the wearables, nor do they publish the kind of rigorous research sleep experts need to establish the credibility of the sleep reports they produce.

The findings of this systematic literature review about Fitbit Charge 4, Garmin Vivosmart 4, and WHOOP are based on our recent comprehensive search of databases for relevant published articles. The included research studies have certain limitations. For example, several investigations evaluating the sleep-tracking capabilities of these wearables involved a relatively limited number of participants, potentially impacting the generalizability of the results. Efforts were made to get additional information about the sample sizes and participants directly from the authors of some included studies. Unfortunately, despite our attempts to contact the authors, we did not receive a response. It is crucial to address these issues transparently, as they may impact the generalizability of our findings. Small sample sizes can introduce variability and limit the statistical power of the findings, underscoring the need for larger and more diverse cohorts to validate the devices’ accuracy across different demographics and sleep conditions. Additionally, it is essential to consider that most of the studies have been conducted in controlled environments, namely sleep laboratories. Although this controlled setting allows for precise data collection and monitoring, it may not fully reflect the real-world sleep experiences of individuals in their natural environments [40]. Sleep laboratory conditions may differ substantially from home environments, where factors such as ambient light, noise, and personal sleep habits can vary widely [41]. Therefore, the generalizability of the findings from laboratory studies to everyday scenarios should be approached with caution. In addition to acknowledging the potential limitations associated with conducting studies in controlled sleep laboratory environments, it is important to recognize the presence of the “first-night effect” in both PSG and wearable sleep-tracking technologies. The first-night effect refers to the phenomenon where an individual’s sleep patterns and quality may be altered due to the unfamiliarity with the sleep-monitoring setup, regardless of whether it occurs in a sleep laboratory or at home [42,43]. This phenomenon is not exclusive to sleep laboratories as it extends to home environments where individuals may experience similar disruptions during the initial adaptation to sleep-tracking devices [44,45]. Although some wearable users may initially find it uncomfortable or unfamiliar to wear a device on their wrist while sleeping, which could potentially impact their sleep quality on the first night of use, there is not a widely recognized first-night effect associated with the use of wrist-worn wearables [46]. In addition, the data of PSG and wearables were collected each time under the same circumstances and environmental factors either in a sleep laboratory or in the home environment, making the first-night effect less relevant. Another notable aspect of the studies is the variations in the duration of the validation of the wearables. The diverse durations across studies are likely due to practical constraints or differences in research protocols. Our decision to include studies with varying protocols was motivated by the aim to include as many relevant papers as possible in our comprehensive review, considering the scarcity of literature on this topic. Future research endeavors should strive for standardized protocols including larger sample sizes to enhance the comparability and power of the results across studies.

Despite these limitations, the findings of this review indicate that the devices with higher relative agreement and sensitivities for multistate sleep (ie, Fitbit Charge 4 and WHOOP) seem appropriate for deriving suitable estimates of sleep parameters and could be used to monitor sustained, meaningful changes in sleep architecture (ie, time spent in different stages of sleep). However, analyses regarding the multistate categorization of sleep (as a specific sleep stage or wake) indicate that all devices can benefit from further improvement. Providers are continuously developing new versions and variants of wearables, which present difficulties for those undertaking independent validation studies. Nevertheless, it can be reasonably assumed that newer models from the same provider will perform at least as well, if not better, than older models when compared against the relevant gold standards [22]. However, although the wearable technology market keeps developing wearable devices, the scientific research on these wearables against PSG remains considerably limited. This scarcity in literature not only reduces our ability to draw definitive conclusions but also highlights the need for more targeted research in this domain. Therefore, the data presented here should not be considered obsolete when the models analyzed are superseded by newer models. Instead, these data should serve as the best approximation of the expected performance of any subsequent models that may be released.

## Supplementary material

10.2196/52192 Multimedia Appendix 1 Search string. In adherence with the PRISMA (Preferred Reporting Items for Systematic Reviews and Meta-Analyses) statement, a comprehensive search of the PubMed, Web of Science, Google Scholar, Scopus, and Embase databases was conducted as shown in the search string.

10.2196/52192 Multimedia Appendix 2 Normative values of sleep parameters. These normative values in healthy male and female individuals of different age groups (mean and SD), derived from the widely recognized Rechtschaffen and Kales scoring system of polysomnography, can provide an additional benchmark when delving into the comparative analysis of sleep parameters measured by wearables [47].

10.2196/52192 Checklist 1 PRISMA (Preferred Reporting Items for Systematic Reviews and Meta-Analyses) checklist.

## Abbreviations

DS: deep sleep
EEG: electroencephalogram
LS: light sleep
PPG: photoplethysmography
PRISMA: Preferred Reporting Items for Systematic Reviews and Meta-Analyses
PSG: polysomnography
REM: rapid eye movement
TST: total sleep time
