# Supplementary material for: Accuracy of Fitbit Charge 4, Garmin Vivosmart 4, and WHOOP Versus Polysomnography: Systematic Review
Source: JMIR Mhealth Uhealth. 2024 Mar 27;12:e52192. doi: 10.2196/52192 (PMC11004611; doi:10.2196/52192)
Supplement: Multimedia Appendix 1 [file mhealth-v12-e52192-s001.pdf]

Multimedia Appendix 1: Search string

“wearable" OR "wristband” OR "sleep tracker" OR "sleep-tracking"	AND	“Whoop" OR "Fitbit Charge 4" OR "Garmin Vivosmart 4”	AND	"validity" OR "accuracy" OR "assessment" OR "performance"	AND	“polysomnography”
