# Supplementary material for: Accuracy of Fitbit Charge 4, Garmin Vivosmart 4, and WHOOP Versus Polysomnography: Systematic Review
Source: JMIR Mhealth Uhealth. 2024 Mar 27;12:e52192. doi: 10.2196/52192 (PMC11004611; doi:10.2196/52192)
Supplement: Multimedia Appendix 2 [file mhealth-v12-e52192-s002.pdf]

Multimedia Appendix 2: Normative values of sleep parameters

	20-39 years		40-59 years		60-95 years	
	**Males (n = 29)**	**Females (n = 30)**	**Males (n = 27)**	**Females (n = 30)**	**Males (n = 34)**	**Females (n = 39)**
**Total Sleep Time (min)**	420 ± 34	433 ± 29	407 ± 37	409 ± 36	375 ± 48	378 ± 50
**Sleep stage 1 (min)**	31 ± 15	27 ± 11	47 ± 16	38 ± 18	62 ± 29	45 ± 18
**Sleep stage 2 (min)**	225 ± 29	226 ± 34	235 ± 39	223 ± 39	207 ± 50	207 ± 45
**Sleep stage 3 (min)**	34 ± 13	32 ± 12	29 ± 9	34 ± 13	26 ± 22	37 ± 19
**Sleep stage 4 (min)**	41 ± 20	51 ± 23	16 ± 16	26 ± 24	13 ± 19	21 ± 22
**REM sleep (min)**	89 ± 16	98 ± 20	81 ± 22	88 ± 25	66 ± 24	68 ± 22
